# Osmoregulation by the gastro-intestinal tract of marine fish at depth – implications for the global carbon cycle

**DOI:** 10.1242/jeb.249834

**Published:** 2025-07-18

**Authors:** Martin Grosell, Bret Marek, Sarah Walls, Carolyn Pope, Cameron Sam, Rachael M. Heuer, Amanda M. Oehlert

**Affiliations:** ^1^ Department of Marine Biology and Ecology, Rosenstiel School of Marine, Atmospheric, and Earth Science, University of Miami, 4600 Rickenbacker Causeway, Miami, FL 33149-1098, USA; ^2^ Department of Marine Geosciences, Rosenstiel School of Marine, Atmospheric, and Earth Science, University of Miami, 4600 Rickenbacker Causeway, Miami, FL 33149-1098, USA

**Keywords:** Blackbelly rosefish, Mesopelagic fish, Ichthyocarbonate, Osmoregulation, Intestinal fluid chemistry

## Abstract

Marine fish are likely one of the top producers of biogenic carbonate in the oceans. However, nothing is known about the production rate and composition of intestinal carbonate (ichthyocarbonate) excreted by mesopelagic fishes, which are small, fragile and account for up to 94% of global fish biomass. To address this knowledge gap, and associated uncertainty of global ichthyocarbonate production, we identified a model species residing at 350–430 m, depths relevant for mesopelagic fishes. The blackbelly rosefish (*Helicolenus dactylopterus*) lacks swim bladders and survives capture and transfer to the lab. Freshly collected blackbelly rosefish, maintained at 6°C, contained high amounts of intestinal ichthyocarbonate (0.4 g kg^−1^) and excreted ∼5 mg kg^−1^ h^−1^ ichthyocarbonate, in agreement with expectations based on allometric and thermal relationships for other species. Despite longer intestinal residence time, intestinal and excreted ichthyocarbonates are similar in crystallite morphology, composition and sinking rate, but have a higher dissolution rate than that produced by shallow water species at higher temperatures, ruling out strong effects of pressure and low temperatures on ichthyocarbonate formation and excretion. Considering allometric and thermal relationships, the metabolic rate of blackbelly rosefish is lower than that of other marine fish in general, and mesopelagic fishes in particular. Our observations support assumptions of ichthyocarbonate excretion by mesopelagic fishes, and suggest that thermal and allometric relationships for ichthyocarbonate excretion determined from shallow water species extend to fish populations at depth.

## Teaser

**Summary:** Blackbelly rosefish at greater depths contribute to global carbonate production according to predictions from size and temperature. Pressure and temperature have little effect on ichthyocarbonate characteristics.

## INTRODUCTION

Since the first recognition that carbonate produced by marine fishes, ‘ichthyocarbonate’, forms a significant contribution to the marine inorganic carbon cycle (Wilson et al., 2009), global fish biomass estimates have increased owing to the recent realization of a large biomass of mesopelagic fishes (Oehlert et al., 2024a). Linear scaling of global ichthyocarbonate production rates using updated global fish biomass estimates revealed that marine fishes may be the top contributor to oceanic carbonate production (Oehlert et al., 2024a). This re-evaluation assumes that mesopelagic fishes produce ichthyocarbonate and that excretion rates are similar to those measured for shallow water species. However, these two assumptions have yet to be tested.

The first assumption of ichthyocarbonate production is well justified. Ichthyocarbonates have been observed in all osmoregulating marine fishes examined to date (Smith, 1930; Gregorio et al., 2013; Grosell, 2006; Grosell et al., 2001; Grosell and Oehlert, 2023; Walsh et al., 1991). Ichthyocarbonate formation is critical for survival because osmotic pressure reduction in the intestinal lumen facilitates water absorption (Grosell et al., 2009; Grosell and Oehlert, 2023; Wilson et al., 2002). The second assumption is perhaps less certain for the following reasons. First, fish at mesopelagic depths experience lower environmental temperatures and thus will have correspondingly lower metabolic rates, which are expected to yield lower ichthyocarbonate excretion rates (Ghilardi et al., 2023; Wilson et al., 2009). Second, fish at depth maintain high plasma trimethylamine oxide (TMAO) concentrations to prevent denaturing of proteins resulting from high pressure (Yancey et al., 2014). Thus, they have higher osmotic pressure, likely allowing for lower drinking rates and therefore lower ichthyocarbonate production. Conversely, mesopelagic fishes are generally small and may therefore be expected to have higher ichthyocarbonate excretion rates as a function of metabolic scaling, an important factor for shallow water subtropical and tropical fishes (Salter et al., 2018). Furthermore, mesopelagic environments are characterized by low aragonite saturation state (Ω; Jiang et al., 2015), interpreted to be driven by high aerobic respiration, which remineralizes sinking organic matter and converts it to dissolved inorganic carbon, which elevates partial pressure of CO_2_ (*P*_CO_2__) in deep water masses (Carter et al., 2014; Kwon et al., 2009; Sarmiento and Gruber, 2013). Elevated seawater *P*_CO_2__ is a factor known to increase ichthyocarbonate production rates in controlled experiments (Gregorio et al., 2019). Ichthyocarbonate excretion by mesopelagic fishes may thus be higher than otherwise expected. Further, extensive vertical migration by many mesopelagic fishes (Govindarajan et al., 2023) may be associated with higher metabolic and ichthyocarbonate excretion rates. Finally, nothing is known about the morphology, mineralogy, organic matter content and fate of ichthyocarbonate that may be excreted by mesopelagic fishes. A previous study of ichthyocarbonate production by temperate species at temperatures as low as 10°C suggested that mineralogy, composition and morphology are similar to those of subtropical and tropical species (Salter et al., 2019), but we are aware of no studies considering ichthyocarbonates produced at deep water pressure conditions or at temperatures below 10°C.

If the proposed relationship between metabolic rate and ichthyocarbonate production rate holds true, estimates of ichthyocarbonate excretion rates by mesopelagic fishes could be made from metabolic rate measurements. Yet little is known about mesopelagic fish physiology because it is challenging to obtain intact individuals and maintain them at sea surface pressure. Trawling is the primary method of collecting mesopelagic fishes. The inevitable temperature increase and pressure decrease experienced by captured individuals leaves them compromised on arrival to the sea surface. Oxygen consumption rates of surviving individuals have been measured shortly after capture (Donnelly and Torres, 1988; Torres and Somero, 1988), but mesopelagic specimens were all affected by collection, survived only 1–3 days in the lab, and thus may not fully reflect metabolic rates of healthy individuals.

There is a clear need to explore production and excretion rates, composition and fate of ichthyocarbonate by mesopelagic fish collected from 200–1000 m depth and at temperatures less than 10°C. The blackbelly rosefish (*Helicolenus dactylopterus*) presents a unique opportunity to overcome these challenges. After hook and line collection, blackbelly rosefish do not appear to suffer from pressure effects when brought to the surface from ∼400 m, likely because they lack swim bladders. With temperature maintained at 6°C during transport and in the laboratory, blackbelly rosefish survive for weeks, and several individuals fed in captivity. Thus, although not a vertically migrating species, blackbelly rosefish provide a rare model for studying the composition, production rate and fate of ichthyocarbonate, alongside measurements of metabolic rate in fish living in the same environmental conditions as mesopelagic fishes.

We aimed to determine whether ichthyocarbonate precipitates are present in the intestine of fish from ∼400 m, and examine their chemistry, crystallite structure and organic matter content, as well as the composition of blood plasma and intestinal fluids. Further, we tested the hypothesis that blackbelly rosefishes excrete ichthyocarbonate, albeit at low rates, and sought to determine whether environmental pressure affects ichthyocarbonate composition and fate. We also tested the hypothesis that metabolic rate in lab-acclimated, healthy individuals would differ from previous studies of mesopelagic fishes collected in trawls. Production and excretion of ichthyocarbonate by blackbelly rosefish was confirmed, thus measurements of ichthyocarbonate size, crystallite morphology, chemical composition and specific gravity were conducted. In concert, these measurements enabled the first estimates of sinking and dissolution rates, allowing predictions of the fate of ichthyocarbonate excreted by fish residing at mesopelagic depths.

## MATERIALS AND METHODS

Blackbelly rosefish [*Helicolenus dactylopterus* (Delaroche 1809)] were collected using hook and line between 350 and 430 m using 7/0 circle hooks and squid as bait. Fish were brought to the surface using an electric reel taking ∼8 min from hookset to dehooking at the surface. Upon collection, fish were either sampled immediately or transferred to a cooler containing aerated seawater maintained at ∼6°C. In the latter case, fish were transported to similarly chilled tanks at the Rosenstiel School of Marine, Atmospheric and Earth Science, Miami, FL, USA. All procedures involving animals were approved by the institutional animal care and use committee (IACUC) under protocol number 22–150.

### Sampling freshly collected fish

Blood was sampled into heparinized 1 ml syringes fitted with 22-gauge needles via caudal puncture, transferred to a bullet tube, and stored on ice until processing. Subsequently, intestinal contents from the anterior and posterior segments were collected via dissection of euthanized fish and samples were transferred into bullet tubes. Fish were then sexed and weighed, and a muscle sample was collected.

### Experiments on fish in captivity

In the lab, fish were maintained in groups of one to three in 70-liter tanks with aerated and biofiltered seawater at 6°C, and 75% water changes were performed daily. Fish (132–551 g) were offered squid once weekly. No mortality of captive fish occurred. Temperature, salinity, dissolved oxygen and pH were recorded daily, and water samples were collected prior to water changes for ammonia analysis.

The tank bottoms were siphoned daily and excreted ichthyocarbonates were separated manually from fecal matter using disposable transfer pipettes. Metabolic rates of individual fish, fasted for >7 days, were determined over at least 48 h by automated intermittent flow respirometry using Loligo respirometers and a Vitrox system operated by Autoresp software. Two respirometer sizes of 1.08 and 22.6 liters were used depending on the size of the fish. Before and after each respirometry trial, background O_2_ consumption was determined in empty respirometers and metabolic rates were corrected for background. After a period of ichthyocarbonate production rate measurements and completion of respirometry, the captive fish were anesthetized using buffered MS-222 and sampled as outlined above for freshly collected fish.

Prompted by observations on high osmotic pressure in blood plasma and intestinal fluids from field-collected fish (the potential impacts of angling stress on blackbelly rosefish), a subset (*n*=9) of captive fish were subjected to a simulated angling protocol at the end of the respirometry experiments. Each fish was transferred from the respirometers to a bucket containing seawater at 6°C, and shortly after to a bucket containing 10°C seawater where the fish was chased manually for 2 min, then transferred to a bucket containing 15°C seawater, and again chased for 2 min. This procedure was repeated at 20 and 25°C. After the 2-min chase in 25°C, a blood sample was obtained via caudal puncture, and the fish was returned to 6°C for recovery. Fish were allowed to recover from the simulated angling stress for a minimum of 4 days, during which they were not fed, before being sampled to obtain blood, intestinal fluids, intestinal ichthyocarbonates and a muscle sample as described above. Temperature interval selection and exposure duration were based on temperature–depth profiles in the Straits of Florida (Meinen and Luther, 2016) and the 8 min angling duration experienced for field-collected fish.

### Analytical procedures

Blood hematocrit was determined using heparin-coated glass capillary tubes, and plasma was obtained after centrifugation (6000 ***g*** for 5 min). Intestinal content was allowed to settle, and intestinal fluids were pipetted off, leaving ichthyocarbonate and any fecal samples in the original bullet tubes. Plasma and intestinal fluids were analyzed for osmotic pressure by a vapor pressure osmometer (Wescor VAPRO 5520) and total CO_2_ (TCO_2_) using a Corning 965 total CO_2_ analyzer. Plasma cations and anions were analyzed after appropriate dilutions using flame atomic absorption (Varian 220FS) and anion chromatography (DIONEX Integrion HPIC with an AS-AP autosampler), respectively.

Average ichthyocarbonate excretion rates (mg h^−1^ kg^−1^) were determined from the mass collected, time since last collection, and fish mass for each tank. Routine metabolic rates were determined from all background-corrected metabolic rate measurements, except those collected the first 3 h after the fish were placed in respirometers. Standard metabolic rate (SMR) was determined using the double Gaussian method (Frank et al., 2023).

Ichthyocarbonates, obtained from the intestine via dissection or siphoned from tank bottoms, were sorted in seawater to remove any undigested food or parasites and were photographed for size analysis using Fiji (Image J2 Version 2.14.0/1.54f). Ichthyocarbonates were then used in measurements of dissolution rate, specific gravity, Ca^2+^ and Mg^2+^ concentrations, total organic carbon content (TOC), C/N ratios, total carbonate content or stable carbon isotope ratios of the carbonate (δ^13^C_icthhyo_ values) and organic matter (δ^13^C_org_ values). Subsamples were also fixed for examination using scanning electron microscopy (SEM). Values of δ^13^C_icthhyo_ allow for assessment of the carbon source used to produce the mineral fraction of ichthyocarbonates, which can include ingestion of seawater dissolved inorganic carbon (DIC) and carbon from endogenous CO_2_ ultimately derived from assimilated dietary carbon (Oehlert et al., 2024a). We refer to this latter fraction as ‘dietary carbon’ in the following. Dissolution rate measurements were performed by a pH-stat approach (Folkerts et al., 2024) in seawater at 6°C, which allowed for a constant aragonite saturation state (Ω=1) during dissolution rate measurements, equivalent to estimated aragonite saturation state at the location and depth where the fish were collected (Cai et al., 2010). In brief, pH was monitored and maintained constant despite continued dissolution of ichthyocarbonate by addition of HCl via microburettes. Rate of acid addition to maintain constant pH is directly equivalent to dissolution rate (Folkerts et al., 2024). Statistical change point analyses conducted in RStudio using the Rbeast package (Zhao et al., 2013) were performed on the resulting titration curves, and rates were calculated for one to three phases according to the changepoint analysis. Ichthyocarbonate specific gravity was determined by the neutral buoyancy approach (Folkerts et al., 2024; Pasparakis et al., 2022).

For measurements of ichthyocarbonate δ^13^C_icthhyo_ and δ^13^C_org_ values, concentrations of Ca^2+^, Mg^2+^ and TOC, ichthyocarbonates were rinsed briefly in nanopure water and dried at room temperature. A subset of the dried ichthyocarbonate samples was analyzed using a Kiel and Delta Plus in the Stable Isotope Laboratory (SIL) at the Rosenstiel School to determine δ^13^C_ichthyo_ values (reported in ‰ V-PDB) as described previously (Oehlert et al., 2024a), and another subset digested in trace metal grade nitric acid prior to analysis of [Ca^2+^] and [Mg^2+^] via flame atomic absorption (Varian 220FS) as described previously (Folkerts et al., 2024). Finally, another subset was acidified using 5% HCl to remove carbonate, filtered and analyzed for total organic carbon content (TOC), C/N ratios and δ^13^C_org_ values (reported in ‰ V-PDB) using a Delta V Advantage or Delta Q as previously described (Oehlert et al., 2024a,b). Carbonate content per wet mass of ichthyocarbonate was determined by mass loss after acid digestion and filtration. Contributions of dietary carbon and seawater DIC were determined using IsoError (Phillips and Gregg, 2001) as previously conducted (Oehlert et al., 2024a). Model inputs include our new measurements of δ^13^C_icthhyo_ values and muscle δ^13^C values as a proxy for diet composition after correction for assimilation (+1‰; Deniro and Epstein, 1978). Model estimates of 400 m seawater δ^13^C_DIC_ values from the North Atlantic (−0.5‰; Liu et al., 2021) parameterized the seawater carbon source. Epsilon (ε) values for mineralogical fractionation were parameterized using the equation for synthetic high magnesium calcite (Jimenez-Lopez et al., 2006) using measurements of mol%MgCO_3_ from blackbelly rosefish ichthyocarbonate (Fig. 4). Results are reported as average dietary carbon contribution (±95% confidence intervals following Philips and Gregg, 2001) to ichthyocarbonate.

For SEM analyses, ichthyocarbonate samples collected from the intestines were fixed with 2% glutaraldehyde in Ca- and Mg-free PBS, whereas tank-collected ichthyocarbonate was preserved in 2% glutaraldehyde in 0.05 mol l^−1^ sodium cacodylate buffered and filtered seawater upon collection. Both types were dehydrated through a graded ethanol series (30%, 50%, 70%, 100%) and dried in three changes of hexamethyldisiloxane (HMDS). Samples were carbon-coated and imaging was conducted in a field emission SEM (JEOL JSM 7100F SEM) at BioNIUM labs at the University of Miami.

### Statistical analysis

Plasma ion concentrations, osmotic pressure, TCO_2_ and hematocrit in field- versus lab-collected blood samples, including samples collected pre- and post-simulated angling stress, were compared using a two-way ANOVA with lab versus field and angling stress (including simulated) versus rest followed by Bonferroni-corrected comparisons of individual means. Holm–Šidák tests were used in cases that failed normality tests. Parameters from intestinal fluids from anterior and posterior regions of field- and lab-collected samples were compared by two-way ANOVAs followed by Bonferroni-corrected comparisons of individual means. Again, Holm–Šidák tests were used in cases that failed normality tests. In all cases, *P*<0.05 was assumed to indicate statistically significant differences. In the text, we refer to data as means±s.e.m. with *n*-values listed in the figure legends.

## RESULTS

**Fig. 1.**
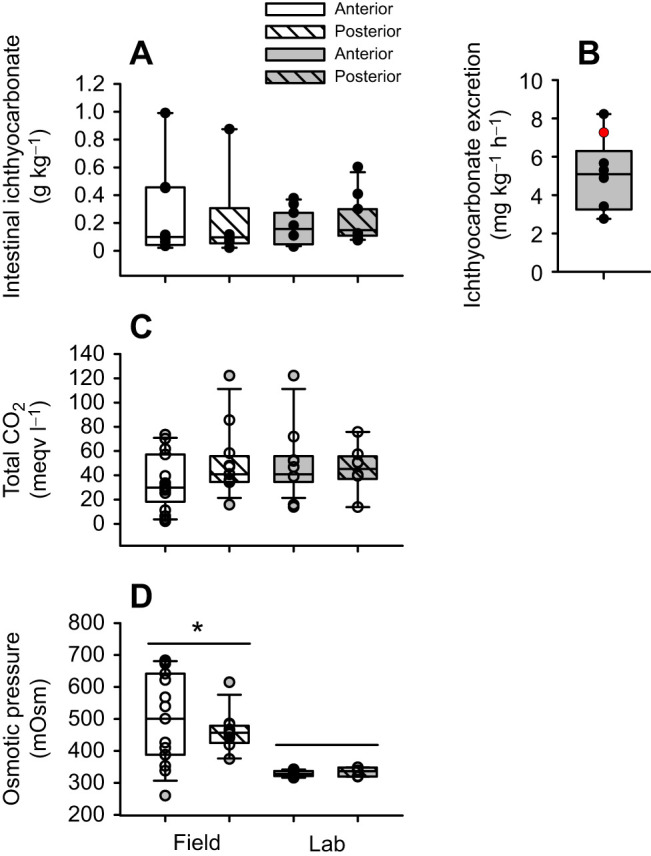
**Intestinal fluid characteristics and ichthyocarbonate excretion rates of blackbelly rosefish.** (A) Presence of ichthyocarbonate in the intestinal lumen of field-collected (*n*=6–8) and lab-acclimated (*n*=11) blackbelly rosefish and (B) ichthyocarbonate excretion rate by lab-acclimated blackbelly rosefish (*n*=6 tanks, with 1–2 fish per tank for a total of 168 days of collection) assessed by collecting and quantifying ichthyocarbonates accumulated in the holding tanks over 24 h periods. Red symbol shows the expected excretion rate for the size of fish used in the present study and at 6°C based on the relationship used to derive original global estimates of ichthyocarbonate excretion by Wilson et al. (2009). (C) Concentration of total CO_2_ in intestinal fluids obtained from field-collected (*n*=12–17) and lab-acclimated (*n*=8–10) blackbelly rosefish. (D) Osmotic pressure of intestinal fluids obtained from field-collected (*n*=6–9) and lab-acclimated (*n*=7–10) blackbelly rosefish. Symbols represent individual observations; box plots show median values and confidence intervals. *Statistically significant differences between field and corresponding lab collected values.

Of 13 angled blackbelly rosefish, 12 survived and no mortality was observed after arrival to the lab. Total ammonia concentration in the holding tanks prior to daily water changes was 49±7 µmol l^−1^. Both anterior and posterior segments of the intestine in field-collected as well as lab-acclimated blackbelly rosefish contained ichthyocarbonate at masses of 0.28±0.12, 0.21±0.13, 0.17±0.12 and 0.22±0.05 g kg^−1^, respectively, with no significant differences among groups (Fig. 1). Lab-acclimated fish excreted ichthyocarbonate at a rate of 5.04±0.78 mg kg^−1^ h^−1^ (Fig. 1), comparable to expectations from the relationship between fish size and temperature presented previously (Wilson et al., 2009). As expected, intestinal fluids were rich in total CO_2_, with mean concentrations of 34.6±5.5 and 49.9±8.2 meqv HCO_3_^−^ l^−1^ in the anterior and posterior intestine of field sampled fish, respectively. Values from lab-acclimated fish were 48.5±4.6 and 45.5±6.3 meqv HCO_3_^−^ l^−1^ in the anterior and posterior intestine, respectively, and not different from the field-collected values (Fig. 1). Field-collected blackbelly rosefish had significantly higher osmotic pressure of 497±37 and 457±18 mOsm in the anterior and posterior intestinal fluids, respectively, compared with 329±3 and 333±5 mOsm observed in lab-acclimated fish. There were no differences between anterior and posterior intestinal fluid osmotic pressure in either field-sampled or lab-acclimated fish (Fig. 1).

Intestinal fluid composition (Table 1) in blackbelly rosefish acclimated to lab conditions had characteristics similar to those reported for other species, including relatively low [Na^+^], low [Cl^−^], elevated [Mg^2+^] and [SO_4_^2−^] and low mM concentrations of K^+^ and Ca^2+^ (Larsen et al., 2014; Marshall and Grosell, 2006). However, distinct from other species, no differences were observed between the anterior and posterior segment of the intestine from lab-acclimated fish, although [Mg^2+^] and [SO_4_^2−^] tended to be higher and [Ca^2+^] tended to be lower in posterior segments as seen in other species (Larsen et al., 2014). In contrast, anterior intestinal fluids from field-collected Blackbelly rosefish had significantly higher Na^+^, Cl^−^ and Ca^2+^ concentrations than in corresponding fluids from lab-acclimated fish and had lower Mg^2+^ concentrations. Further, both Na^+^ and Cl^−^ concentrations were higher in anterior than in posterior fluids from field-collected fish (Table 1).

**Table 1.** Ionic composition (mmol l^−1^) of fluid in the anterior and posterior intestine of field-collected and lab-acclimated fish

	Field-collected		Lab-acclimated	
Ion	Anterior	Posterior	Anterior	Posterior
[Na^+^]	128.9±28.5 (10)*	72.6±14.6 (10)^‡^	35.1±5.4 (11)	61.1±13.5 (9)
[Cl^−^]	165.9±26.1 (10)*	105.3±14.0 (10)^‡^	104.2±9.2 (11)	124.6±17.6 (9)
[K^+^]	4.0±0.7 (10)	5.2±0.8 (10)	4.1±0.6 (11)	3.0±0.8 (9)
[Ca^2+^]^a^	7.8±1.6 (10)	9.7±1.3 (10)	3.8±0.6 (11)	2.8±0.8 (9)
[Mg^2+^]^a^	96.7±14.8 (10)	124.4±13.7 (10)	162.2±25.3 (11)	173.3±17.7 (9)
[SO_4_^2−^]^b^	51.8±7.8 (10)	71.5±7.5 (10)	83.4±12.4 (11)	90.6±11.5 (9)

Data are means±s.e.m. (*n*). ^a^Overall statistically significant difference between field-collected and lab-acclimated fish. ^b^Overall difference between anterior and posterior. *Difference from corresponding lab-acclimated samples. ^‡^Difference from corresponding anterior samples.

**Fig. 2.**
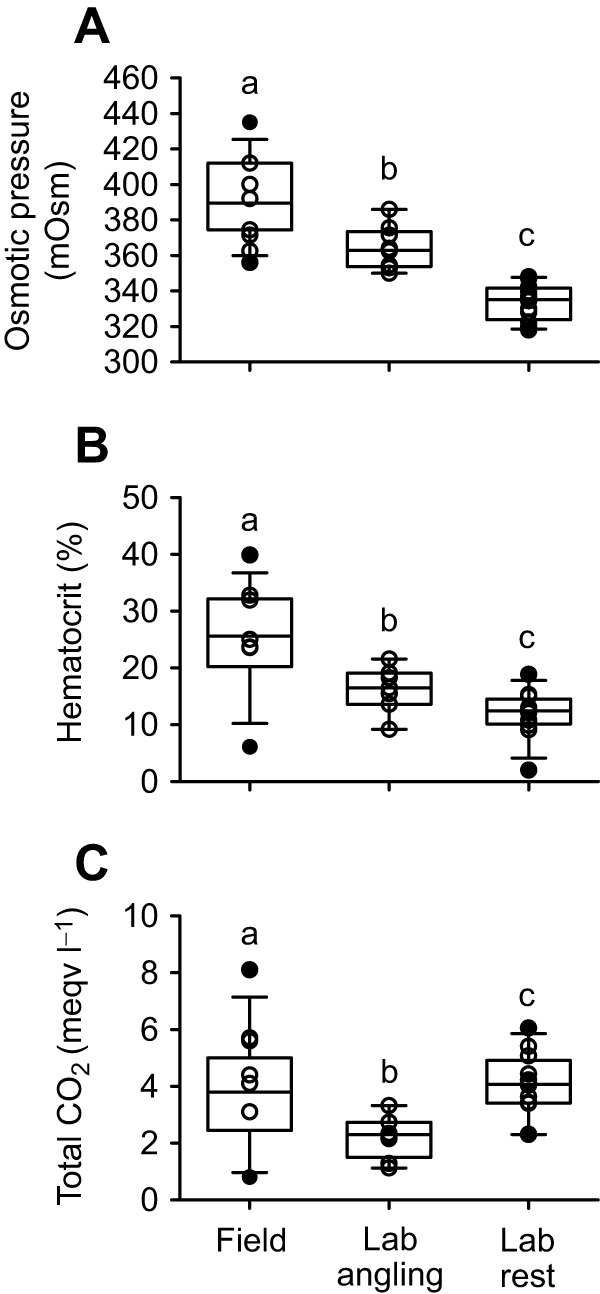
**Blood plasma characteristics for blackbelly rosefish.** Plasma osmotic pressure (A, *n*=9–15), hematocrit (B, *n*=7–14) and plasma total CO_2_ (C, *n*=8–13) in blackbelly rosefish sampled in the field immediately after capture, from lab-acclimated fish after simulated angling and from lab-acclimated fish at rest. Open symbols represent individual observations; box plots show median values and confidence intervals. Black symbols show identified outliers. Bars labelled with different letters are statistically different. Groups were compared in a one-way ANOVA with appropriate *post hoc* analyses. A paired *t*-test was used for simulated angling versus rest in lab-acclimated fish, *P*<0.05.

Blood osmotic pressure was elevated in field-collected blackbelly rosefish (391±6 mOsm) compared with lab-acclimated fish at rest (334±3 mOsm), and was higher than that of lab-acclimated fish subjected to simulated angling stress (364±4 mOsm). Further, plasma osmotic pressure in lab-acclimated fish subjected to simulated angling stress was higher than that in lab-acclimated fish at rest (Fig. 2). A similar pattern was observed for blood hematocrit, with field-collected fish (25.1±2.3%) hematocrit higher than that of both lab-acclimated fish at rest (11.9±1.2%) and lab-acclimated fish exposed to simulated angling stress (16.2±1.5%) (Fig. 2). In contrast, plasma TCO_2_ levels were similar in field-collected (3.8±0.5 meqv HCO_3_^−^) and lab-acclimated fish at rest (4.1±0.3 meqv HCO_3_^−^). However, lab-acclimated animals subjected to angling stress displayed significantly reduced TCO_2_ at 2.2±0.3 meqv HCO_3_^−^ (Fig. 2).

Plasma composition of lab-acclimated blackbelly rosefish agreed with results from other marine species, with Na^+^ and Cl^−^ contributing most of the osmotic pressure, low mmol l^−1^ concentrations of K^+^ and Ca^2+^ and sub-mmol l^−1^ concentrations of Mg^2+^ and SO_4_^2−^ (Table 2). No differences were observed in ion concentrations between lab-acclimated fish at rest and after simulated angling. In contrast, field-collected fish showed ion concentrations that were increased overall (Table 2), although the trend escaped statistical significance for Na^+^ and K^+^, accounting for the observed increase in osmotic pressure (Fig. 2).

**Table 2.** Blood ionic composition (mmol l^−1^) in samples collected by caudal puncture from field-collected, lab-acclimated, and lab-acclimated fish subjected to simulated angling stress

Ion	Field-collected	Lab-acclimated	Simulated angling stress
[Na^+^]	192.5±4.3 (10)	188.9±6.4 (12)	188.8±10.8 (9)
[Cl^−^]	196.3±6.8 (10)*	158.7±6.9 (12)	161.1±4.9 (9)
[K^+^]	6.0±1.8 (10)	2.8±0.4 (12)	3.0±0.5 (9)
[Ca^2+^]	3.5±0.2 (10)*	2.4±0.3 (12)	2.1±0.4 (9)
[Mg^2+^]	3.7±0.8 (10)*	0.9±0.1 (12)	0.9±0.2 (9)
[SO_4_^2−^]	1.8±0.4 (10)*	0.2±0.1 (12)	0.3±0.1 (9)

Data are means±s.e.m. (*n*). *Statistically significant difference from corresponding lab-acclimated samples (*P*<0.05).

**Fig. 3.**
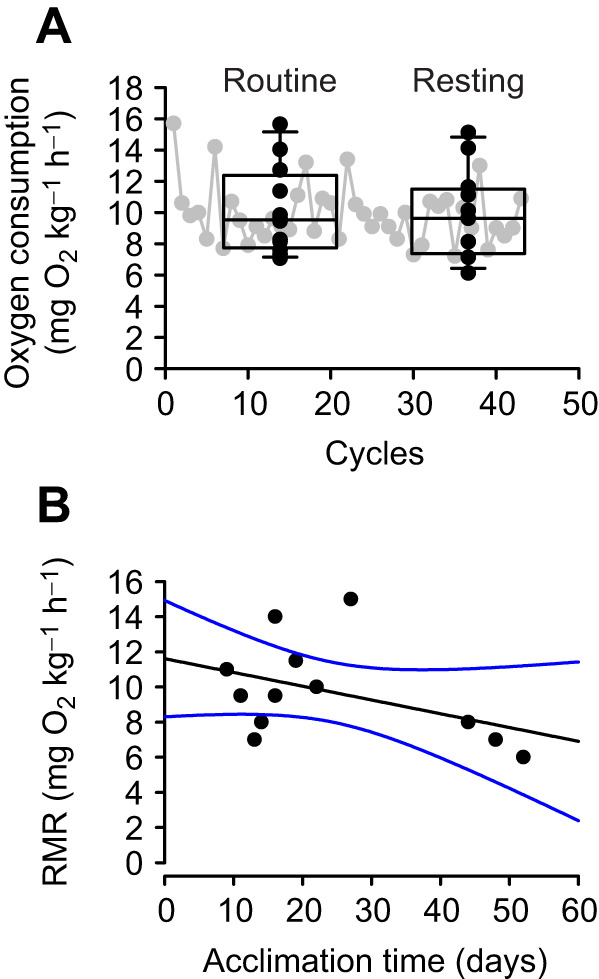
**Oxygen consumption of blackbelly rosefish.** (A) Routine and resting (RMR) oxygen consumption rate by lab-acclimated blackbelly rosefish (*n*=12; black symbols), and a representative trace of oxygen consumption by an individual fish over a 3-day period (gray symbols). The first three cycles were not included in calculations of metabolic rates. (B) Resting metabolic rate (RMR) as a function of days in captivity. Symbols represent individual observations; box plots show median values and confidence intervals.

Mean resting (RMR) and routine metabolic rates of blackbelly rosefish were and 9.7 and 10.0 mg O_2_ kg^−1^ h^−1^, respectively, and oxygen consumption did not change over time after three initial measurements (Fig. 2). Further, RMR showed no significant change over time in captivity between 9 and 52 days post capture (Fig. 3).

**Fig. 4.**
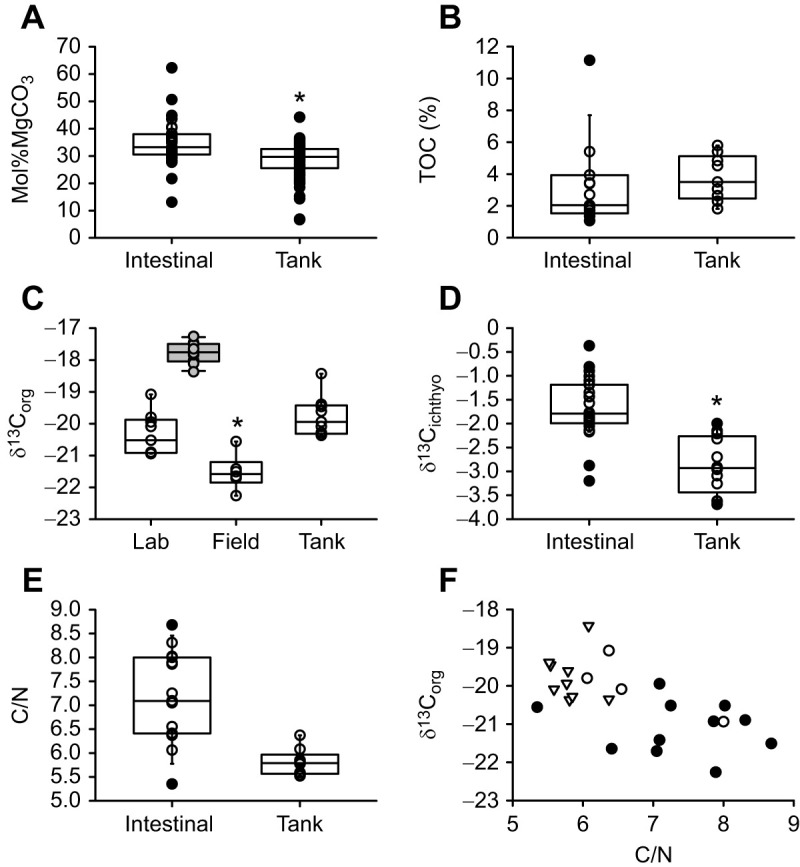
**Characteristics of ichthyocarbonates produced by blackbelly rosefish.** (A) Mol%MgCO_3_ from the anterior and posterior intestine from field-collected and lab-acclimated blackbelly rosefish (*n*=8, 11, 9 and 8, respectively) and mol%MgCO_3_ of ichthyocarbonates collected from the holding tanks (*n*=77). Mol%MgCO_3_ of intestinal ichthyocarbonates was not different between field versus lab-acclimated fish or between anterior and posterior intestinal segments, but overall was higher than that of excreted ichthyocarbonates. (B) Fraction of ichthyocarbonates present as organic carbon (total organic carbon, TOC) in the anterior and posterior intestine from field-collected and lab-acclimated blackbelly rosefish (*n*=1, 1, 2 and 4, respectively) and in icthyocarbonates collected from the holding tanks (*n*=9). No statistically significant differences were observed. (C) Open boxes show δ^13^C_org_ values from ichthyocarbonates from the anterior and posterior intestine of field-collected and lab-acclimated blackbelly rosefish (*n*=4, 5, 2 and 4, respectively) and δ^13^C_org_ values from ichthyocarbonates collected from the holding tanks (*n*=9). The δ^13^C_org_ values did not differ between intestinal segments but were more negative in samples obtained from the intestine of field-collected fish compared with samples obtained from the intestine of lab-acclimated fish and compared with tank-collected samples. δ^13^C values of muscle tissue from field-collected and lab-acclimated fish (*n*=5 in both cases; shown in gray) showed no statistically significant difference. (D) δ^13^C_ichthyo_ values in ichthyocarbonates from the anterior and posterior intestine of field-collected and lab-acclimated blackbelly rosefish (*n*=1, 2, 10, and 16, respectively) and δ^13^C_ichthyo_ values in ichthyocarbonates collected from the holding tanks (*n*=7). δ^13^C_ichthyo_ values of ichthyocarbonates were not different between field-collected versus lab-acclimated fish or between anterior and posterior intestinal segments, but overall were higher in intestinal than in excreted ichthyocarbonates. (E) Molar carbon/nitrogen (C/N) ratios in ichthyocarbonate associated organic matters from the anterior and posterior intestine of field-collected and lab-acclimated blackbelly rosefish (*n*=4, 5, 2 and 4, respectively) and in ichthyocarbonates collected from the holding tanks (*n*=9). C/N ratios of ichthyocarbonate associated organic matter were not different between field versus lab-acclimated fish or between anterior and posterior intestinal segments and intestinal values were not significantly different from excreted ichthyocarbonates. (F) δ^13^C_org_ values as a function of C/N in ichthyocarbonate associated organic matter from intestinal segments (circles) from field-collected (white) and lab-acclimated (black) samples and in ichthyocarbonates collected from the holding tanks (triangles). When statistical tests indicated no significant difference between intestinal segments in field-collected and lab-acclimated fish, the datasets were grouped into one sample type (‘intestinal’) for comparison with tank-collected samples.

Ichthyocarbonate mol%MgCO_3_ did not differ between the anterior and posterior intestine, nor between field-collected and lab-acclimated fish (Fig. 4) with an overall mean of 34.9±1.4%, significantly higher than tank-collected samples (28.4±0.7 mol%MgCO_3_). A similar pattern was observed for δ^13^C_ichthyo_ values, with a mean value for intestinal ichthyocarbonates of −1.69±0.11‰ and higher than −2.88±0.16‰ in tank-collected samples. C/N ratios for intestinal ichthyocarbonates (7.20±0.24) tended to be higher than for tank-collected samples (5.81±0.09), although it escaped statistical significance (Fig. 4). TOC in intestinal ichthyocarbonates also did not differ between intestinal segments or between field versus lab-acclimated fish, with an overall mean of 3.1±0.7%, not distinct from TOC in tank-collected samples (3.8±0.5%; Fig. 4). In contrast, δ^13^C_org_ values differed between field-collected (−19.4±0.04‰) and lab-acclimated (−20.4±0.21‰) fish, although there was no difference between anterior and posterior samples. The δ^13^C_org_ value of tank-collected ichthyocarbonates was −19.80±0.21‰, similar to values for the lab-acclimated intestinal ichthyocarbonates, but higher than observed in field-collected samples (Fig. 4). For reference, δ^13^C of muscle tissue, which did not differ between field and lab-acclimated fish, was −17.8±0.1‰ and more enriched in ^13^C than the organic fraction of any ichthyocarbonate. Results of IsoError models suggest that on average, 28–52% (±7%, 95% CI) of tank-collected ichthyocarbonate and 14–36% (±7%, 95% CI) of intestinal ichthyocarbonate is composed of dietary carbon.

**Fig. 5.**
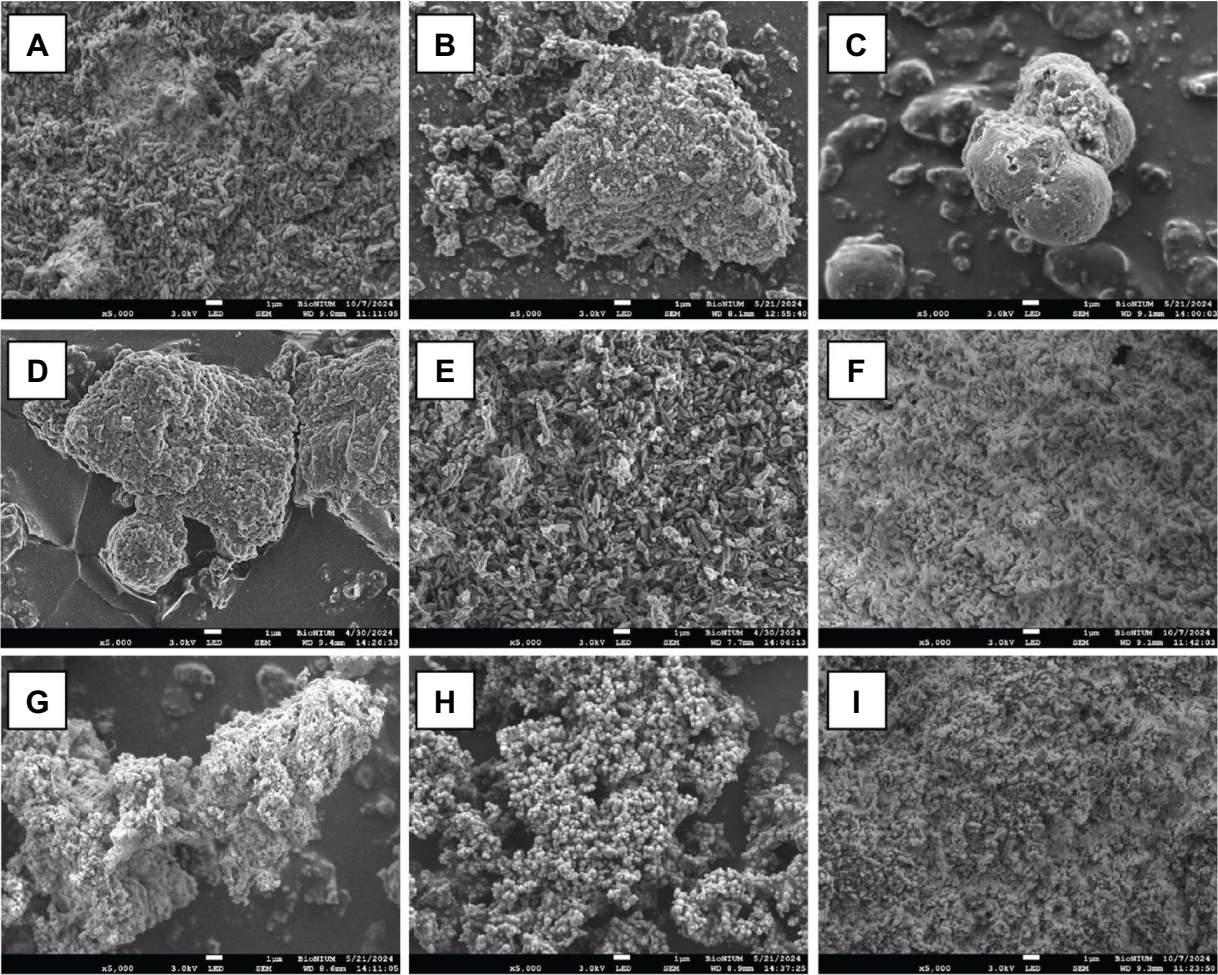
**Scanning electron microscope images of ichthyocarbonate crystallites produced by the blackbelly rosefish at a magnification of 5000× (white scale bars are 1 µm in all images).** Samples were collected from the anterior intestine (A,D,G), posterior intestine (B,E,H) and rectum (C) via dissection. Ichthyocarbonate excreted by the blackbelly rosefish was collected from tank bottoms via siphoning, and crystallite morphology is shown in F and I.

The morphology of ichthyocarbonate crystallites was characterized using SEM as described previously (Salter et al., 2012). Ichthyocarbonate crystallites collected from both segments of the intestine occurred in several morphologies (Fig. 5). In the anterior segment, crystallite morphologies included monocrystalline ellipsoids with lengths between 0.75 and 1.2 µm (Fig. 5A), interlocking, blocky-edged nanospheres (<1 µm) with sheets of organic matter (Fig. 5D), as well as nanospheres (∼0.25 to 0.5 µm) and irregular nanoparticles with rough surfaces (<0.5 µm) embedded in a web-like organic matrix (Fig. 5G). In the posterior intestine, crystallites occurred as aggregates of interlocking and irregular nanocrystals (Fig. 5B), monocrystalline ellipsoids (Fig. 5E) and nanospheres with varying radii (Fig. 5H). Although rare, ∼500 nm nanospheres were also observed to occur within predominantly monocrystalline ellipsoidal crystallites (Fig. 5E), and some crystallites appeared to exhibit morphologies consistent with intergrown ellipsoids with a common center and perpendicular long axes (Fig. 5E) described previously (Salter et al., 2012). Fig. 5C shows a sample collected from the rectum of a blackbelly rosefish, comprising large spheres approximately 5–6 µm in diameter, which frequently occurred as intergrown, lobate spheres rather than individual crystallites. No consistent trend in crystallite morphology was observed with distance along the intestinal tract; ellipsoids, aggregates of nanocrystalline material, and spherical crystallites each occurred in both segments of different blackbelly rosefish specimens (Fig. 5A–C).

Crystallites in tank-collected ichthyocarbonate were more homogeneous than intestinal ichthyocarbonates (Fig. 5F,I). In most samples examined, crystallites appeared as monocrystalline ellipsoids approximately 0.75 to 1.4 µm in length, with subordinate occurrences of irregular nanocrystalline material (Fig. 5F). Less frequently, crystallites from tank-collected ichthyocarbonate occurred as irregular and angular aggregates of nanocrystalline material (Fig. 5I).

**Fig. 6.**
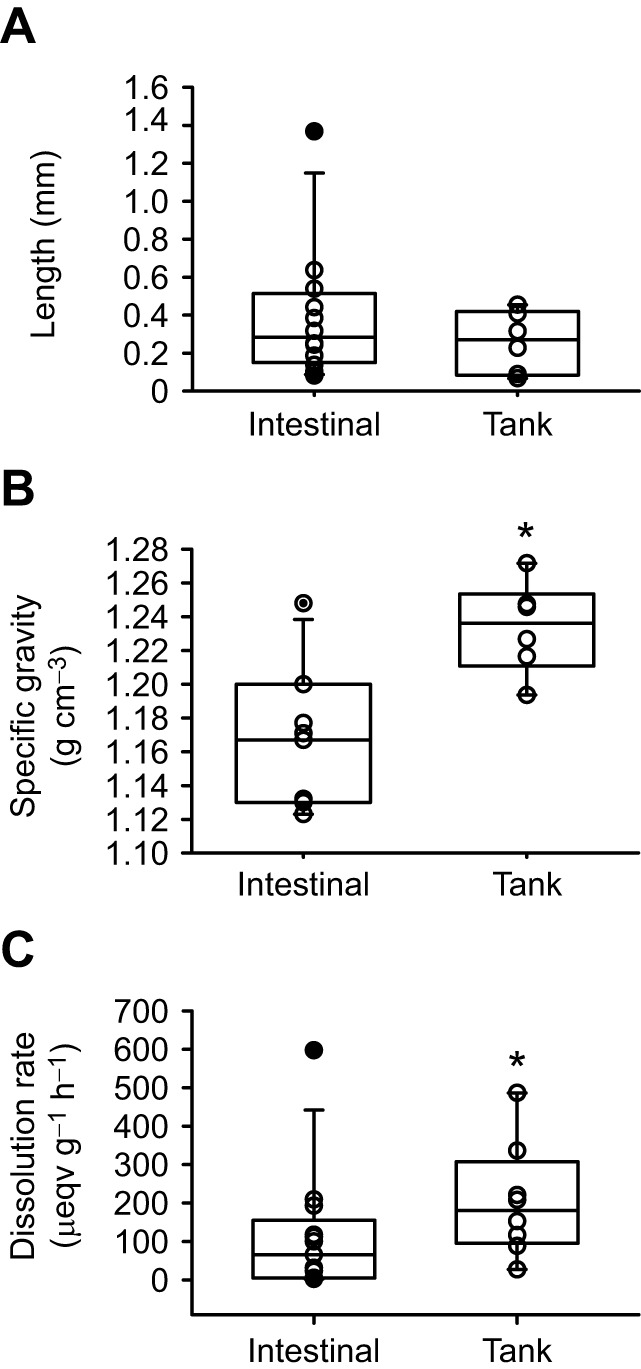
**Parameters relevant for blackbelly rosefish ichthyocarbonate fate.** (A) Average length of individual ichthyocarbonates from the anterior and posterior intestine from three field-collected and three lab-acclimated blackbelly rosefish (mean of 34, 249, 90 and 67, respectively) and of excreted ichthyocarbonates collected from two randomly selected dates from each of three holding tanks (total *n*=352). No statistically significant differences were observed. (B) Specific gravity of ichthyocarbonates from the anterior and posterior intestine from field-collected and lab-acclimated blackbelly rosefish (*n*=4, 3, 2 and 2, respectively) and specific gravity of excreted ichthyocarbonates collected from holding tanks (*n*=7). Specific gravity of intestinal ichthyocarbonates was not different between field-collected and lab-acclimated fish or between anterior and posterior intestinal segments, but overall was lower than that of excreted ichthyocarbonates (*P<0.05). (C) Dissolution rates of ichthyocarbonates collected from the anterior and posterior intestine of field-collected and lab-acclimated blackbelly rosefish (*n*=3 in all cases) and of ichthyocarbonates collected from holding tanks (*n*=8). Dissolution rates of intestinal ichthyocarbonates were not different between field-collected and lab-acclimated fish or between anterior and posterior intestinal segments, but overall were lower than those of excreted ichthyocarbonates (*P<0.05). Open symbols represent individual observations; box plots show median values and confidence intervals. Black symbols show identified outliers.

Intestinal ichthyocarbonates did not differ in length between the anterior and posterior intestinal segments, or between field-collected and lab-acclimated fish, with an overall average length of 0.39±0.10 cm, a value that was not significantly different from the 0.26±0.07 cm of tank-collected ichthyocarbonates (Fig. 6). The specific gravity of ichthyocarbonates collected from the intestinal lumen (1.164±0.012 g cm^−3^) was not different between anterior and posterior segments, or between field-collected and lab-acclimated fish, but was significantly lower than tank-collected ichthyocarbonates (1.234±0.011 g cm^−3^; Fig. 6). Like ichthyocarbonate length and specific gravity, there was no difference in dissolution rates of ichthyocarbonates between intestinal segments or between field-collected and lab-acclimated fish, for an overall mean of 113±45 µeqv HCO_3_^−^ g^−1^ h^−1^. However, the dissolution rate of tank-collected ichthyocarbonates was 205±52 µeqv HCO_3_^−^ g^−1^ h^−1^, significantly higher than that of intestinal samples (Fig. 6).

## DISCUSSION

We demonstrate that at least one species of marine fish collected at depths relevant to the habitat range of mesopelagic fishes osmoregulates (Tables 1 and 2) like other marine teleosts and contains intestinal ichthyocarbonate. When thermal and allometric relationships are considered, excretion rates of ichthyocarbonate (Fig. 1) are comparable to those of shallow water species examined to date. Compositional characteristics of the ichthyocarbonates produced and excreted by blackbelly rosefish are within the range observed for shallow water species at higher temperatures. Further, we demonstrate that the metabolic rate of the benthic blackbelly rosefish is lower than what would be assumed from thermal and allometric relationships for teleosts in general, and lower than reports for other mesopelagic fish that conduct large diel vertical migrations (Belcher et al., 2020).

### Intestinal fluid composition, ichthyocarbonate presence and excretion rates

Both the anterior and posterior intestinal lumen of blackbelly rosefish at 6°C contained ∼0.2 g ichthyocarbonate kg^−1^ fish mass (Fig. 1), for a total of ∼0.4 g ichthyocarbonate kg^−1^ fish mass, substantially higher than the ∼0.02 g ichthyocarbonate kg^−1^ fish mass observed for gulf toadfish at 25°C (Heuer et al., 2016) and seabream at 25°C (Gregorio et al., 2019). Because we observed no difference between intestinal ichthyocarbonate mass recovered from field-collected and lab-acclimated fish, we can rule out an effect of pressure on the amount of ichthyocarbonate found in the intestinal lumen. However, higher ichthyocarbonate content observed in blackbelly rosefish at 6°C suggests that low temperature may drive the difference, possibly owing to longer intestinal retention time. Our observed ichthyocarbonate excretion rates of ∼5 mg ichthyocarbonate kg^−1^ h^−1^ agree with excretion rates predictions (Wilson et al., 2009), when normalized for fish size and temperature used here. However, given the large difference in the amount of ichthyocarbonates contained in the intestine of blackbelly rosefish and gulf toadfish, intestinal ichthyocarbonate residence time must be vastly different. For blackbelly rosefish, turnover time (intestinal ichthyocarbonate mass/ichthyocarbonate excretion rate) is 3.3 days at 6°C, substantially higher than 1.2 days for gulf toadfish at 25°C (Heuer et al., 2016).

Intestinal fluid ionic composition of lab-acclimated blackbelly rosefish was similar to that of other marine teleosts examined to date (Grosell, 2006; Grosell et al., 2001; McDonald and Grosell, 2006; Wilson et al., 1996, 2002), with Na^+^ and Cl^−^ concentrations substantially reduced from seawater levels owing to esophageal and intestinal uptake, and elevated Mg^2+^ and SO_4_^2−^ concentrations arising from intestinal water absorption and divalent ion barrier functions (Table 1). Intestinal fluid TCO_2_ concentrations were high, as expected, but at 35–50 mmol l^−1^ (Fig. 1), these values were at the low end of the range reported for other species sampled at higher temperatures (Grosell, 2006; Grosell et al., 2001; McDonald and Grosell, 2006; Wilson et al., 1996, 2002). The typically low Ca^2+^ concentrations for marine teleost intestinal fluids, a product of ichthyocarbonate formation, were also observed in blackbelly rosefish (Table 1). Lower intestinal fluid TCO_2_ concentrations in blackbelly rosefish did not appear to be influenced by pressure and may thus be a consequence of lower temperature. High intestinal ichthyocarbonate content may explain the relatively low intestinal fluid TCO_2_ concentrations and suggest that more of the HCO_3_^−^ secreted by the intestine is partitioned to the ichthyocarbonate relative to other species. In European flounder (13°C), gulf toadfish (25°C) and seawater-acclimated rainbow trout (11°C), 14, 17 and 14%, respectively, of excreted HCO_3_^−^ equivalents occur as ichthyocarbonate, with the rest as HCO_3_^−^ and CO_3_^2−^ in intestinal fluids (Genz et al., 2008; Wilson et al., 1996, 2002).

### Effects of angling

Intestinal fluid chemistry of field-collected blackbelly rosefish differed from that of lab-acclimated fish by higher Na^+^ and Cl^−^ and lower Mg^2+^ concentrations in the anterior intestine. Collectively, these ion concentration changes account for the increased osmotic pressure observed especially in the anterior, but also the posterior, intestine of field-collected fish (Fig. 1). Although we cannot rule out that the pressure difference between fish at 400 m and those in the lab may affect intestinal fluid chemistry, a more likely explanation is that angling results in the observed changes in intestinal fluid chemistry. As these fish are brought up from 400 m at a speed of ∼1 m s^−1^, some seawater may be pressed into the intestine, which would increase Na^+^, Cl^−^ and Ca^2+^ and decrease Mg^2+^ and SO_4_^2−^ concentrations, as observed (Table 1). We are unaware of any other studies examining the influence of angling stress on intestinal fluid chemistry. The logistical challenges associated with obtaining and holding blackbelly rosefish prevented terminal sampling after simulated angling in lab-acclimated fish, and thus a firm conclusion of the reason for the altered intestinal fluid chemistry cannot be drawn. However, non-terminal blood samples obtained from field-collected fish, lab-acclimated fish and lab-acclimated fish subjected to simulated angling stress offer some insight. Indeed, lab-acclimated fish subjected to simulated angling stress show elevated plasma osmotic pressure (Fig. 2.), an effect also observed in the field-collected fish. Despite our best efforts, the simulated angling did not induce the same magnitude of plasma osmotic pressure disturbance as observed in field-collected fish, possibly a result of angling stress being induced by chasing rather than dragging. Dragging would possibly ventilate the gills to a greater extent, increasing the potential for diffusive ion and water movement across the gills and elevating plasma osmotic pressure. The elevated osmotic pressure in field-collected fish is associated with a significant rise in plasma Cl^−^, Ca^2+^, Mg^2+^ and SO_4_^2−^. Comparing the sum of measured plasma ions with the osmotic pressure, there is no clear evidence for TMAO accumulation in any of the experimental groups. However, because TMAO was not measured directly in the present study and because osmotic coefficients for main plasma ions are not known specifically for these fish, this conclusion should be viewed with caution. Our observations of increased plasma ion concentrations post-angling agree with other reports for marine teleosts (Cooke et al., 2008; O'Toole et al., 2010; Roth and Rotabakk, 2012; Schlenker et al., 2016; Suski et al., 2007). A significant 15% increase in hematocrit (Fig. 2) observed in field-collected fish is likely associated with stress-induced β-adrenergic stimulation of red blood cell (RBC) swelling (Brauner et al., 2002; Herbert and Wells, 2002; Templeman et al., 2014; Wood, 1991). In addition, water loss may also have contributed to the rise in hematocrit. We did not measure hemoglobin, preventing us from determining mean RBC volume, and it remains unclear how much RBC volume changed in the 8 min between hookset and blood sampling. Nevertheless, the fact that all measured ions (tend to) increase in concentration may support the involvement of water loss in hemoconcentration. However, it appears that salt gain also occurred during angling. We observed no change in plasma TCO_2_ in field-collected fish, but a large reduction in lab-acclimated fish subjected to simulated angling (Fig. 2). Disturbance of acid–base balance after exhaustive exercise in fish is well established (Wood, 1991) and is evident from elevated blood lactate levels in marine fish post angling (Cooke et al., 2008; Schlenker et al., 2016; Suski et al., 2007). Reduced TCO_2_ in lab-acclimated fish subjected to simulated angling is consistent with acidosis. However, there was no evidence for acidosis in field-collected fish, potentially because high gill ventilation associated with dragging allowed for effective clearing of excess CO_2_.

### Metabolic rate of blackbelly rosefish – relationship with ichthyocarbonate production

After a 9- to 52-day acclimation to laboratory conditions with holding tanks and respirometers placed in a dark, temperature-controlled room with no other activities, RMR and routine metabolic rates were indistinguishable. The RMR of 9.7 mg O_2_ kg^−1^ h^−1^ at 6°C for blackbelly rosefish was compared with rates predicted from the relationships presented by Killen et al. (2016) of 40.6 mg O_2_ kg^−1^ h^−1^. The relationship between RMR, fish size and temperature presented by Killen and co-workers spans the fish size and temperature used in the present study. However, the data considered by Killen and co-workers include species of different lifestyles, including high activity pelagic species, and show large variation of RMR across species even when normalized for size and temperature, and therefore it is not surprising that the blackbelly rosefish fall below the RMR predicted simply from fish size and temperature. To compare previous reports of oxygen consumption by mesopelagic fish with the current measurements on blackbelly rosefish, we first compared reported metabolic rates with predictions arising from the size and temperature relationship presented by Killen et al. (2016). In a study examining nine species of Antarctic mesopelagic fish (Torres and Somero, 1988), routine metabolic rates were on average 67% of predicted RMRs, whereas the minimum metabolic rates (equivalent to RMR) were on average 38% of predicted RMRs. These metabolic rates for mesopelagics are higher than our observation of RMR for blackbelly rosefish, being 24% of predicted RMR rates. Another study on mesopelagic species from the Gulf of Mexico examined eight species, and found routine oxygen consumption rates to be on average 157% of predicted RMRs, whereas minimal metabolic rates were on average 115% of predicted rates (Donnelly and Torres, 1988). Thus, with attempts to normalize for size and temperature, it appears the blackbelly rosefish have lower metabolic rates than those reported from mesopelagic species. This difference may reflect their benthic lifestyle, the fact that they were not compromised by collection, and that they were fasted and carefully acclimated prior to measurements, or a combination of those factors. The benthic and sedentary lifestyle of blackbelly rosefish admittedly represents a limitation for extrapolation to mesopelagic species that perform vertical migrations and experience higher temperatures on a diurnal basis. Greater activity and higher temperature are both expected to elevate ichthyocarbonate production rates, and the blackbelly rosefish therefore likely is a conservative representation of mesopelagic fish ichthyocarbonate production rates. Future studies ought to explore how behavioral and thermal factors may affect ichthyocarbonate production rates by marine fishes.

The original estimates of global ichthyocarbonate production hinged on the premise that ichthyocarbonate excretion rates reflect metabolic rate, and thus scale with thermal and allometric relationships for fish metabolic rate (Wilson et al., 2009). The rationale was that higher rates of gas exchange would correlate with greater diffusive water exchange at the respiratory surface and thereby correlate with drinking rates which, in turn, correlate strongly with ichthyocarbonate excretion rates (Genz et al., 2008). A general allometric, thermal and lifestyle relationship with ichthyocarbonate excretion has been demonstrated using data from 85 species of reef fishes (Ghilardi et al., 2023). However, after our closer examination, substantial differences among species exist for thermal and allometric relationships (Ghilardi et al., 2023), which may be accounted for by differences in metabolic rates. Thus, we advocate for further measurements linking metabolic rates to ichthyocarbonate excretion rate to refine global ichthyocarbonate production estimates. Here, we found that ichthyocarbonate excretion rates by blackbelly rosefish were 69% of predictions based on thermal and allometric relationships (Wilson et al., 2009), whereas measured metabolic rate of blackbelly rosefish is 24% of predicted values. Thus, it appears that blackbelly rosefish may have higher ichthyocarbonate excretion rates than predicted by metabolic rates compared with what is generally observed. Notably, this conclusion is based on general relationships because blackbelly rosefish is the only species for which metabolic rate and ichthyocarbonate excretion rate have been determined simultaneously. Additional parallel measurements of metabolic and ichthyocarbonate excretion rates are needed to determine whether the relationship between these parameters varies with environmental conditions, allometry, feeding and lifestyle.

### Ichthyocarbonate compositional characteristics

Ichthyocarbonate from blackbelly rosefish is similar in crystallite morphology, magnesium content and geochemical characteristics to that produced by shallow water species, including members of the Scorpaenidae family living in seawater with different temperatures (Table 3). Our new compositional results indicate that the mechanisms of ichthyocarbonate formation, and resulting geochemical characteristics, are likely conserved between fish inhabiting the euphotic and mesopelagic zones of the oceans. Consequently, data for shallow-dwelling fishes provide understanding about the role of marine fish in the carbon cycle that appear extrapolatable to mesopelagic fishes which comprise the majority of global fish biomass.

**Table 3.** Ranges of compositional characteristics of ichthyocarbonate produced by teleost species at variable temperatures

Species	Temperature (^o^C)	Crystallite morphology	Mineralogy (*measured; **inferred)	mol% MgCO_3_	δ^13^C_ichthyo_ (‰)	C/N ratio	TOC (%)	δ^13^C_org_ (‰)	References
*Helicolenus dactylopterus*	6	Monocrystalline ellipsoid, rare nanospheres	HMC**, ACMC**	28.4±0.7	−2.9±0.2	5.8±0.1	3.01±0.7	−19.8±0.2	Present study
*Helicolenus percoides*	10	Monocrystalline ellipsoid	HMC*	28					Salter et al. (2019)
*Pterois volitans*, *Dendrochirus zebra*, *Scorpaenopsis diabolus*	25	Monocrystalline ellipsoid	HMC*	33					Salter et al. (2019, 2018)
*Opsanus beta*	25	Monocrystalline ellipsoid	HMC*	32.3±0.8	−4.2±0.3	7.7±1.6	5.5±2.4	−19.2±1.4	Folkerts et al. (2024); Oehlert et al. (2024a,b); Woosley et al. (2012)
*Notolabrus celidotus*, *Notolabrus fucicola*, *Notothenia angustata*, *Pseudophycis bachus*, *Acanthoclinus fuscus*, *Forsterygion nigripenne*, *Parapercis colias*, *Congiopodus leucopaecilus*, *Genyagnus monopterygius*, *Gastroscyphus hectoris*	10	Ellipsoids, rhombohedron, sphere/dumbbell nanospheres, rare wheatsheaf	HMC, LMC, ACMC*	18–45	**–**	**–**	**–**	**–**	Salter et al. (2019)
*Lates calcarifer*, *Lutjanus russellii*, *Acanthopagrus australis*, Gobiid spp., *Fistularia commersonii*, *Mugil cephalus*, *Atule mate*, *Acanthoclinus ogilbyi*	18	Ellipsoids, rhombohedron, sphere/dumbbell nanospheres, rare wheatsheafs	HMC, LMC, ACMC*	22–34	**–**	**–**	**–**	**–**	Salter et al. (2019)
*Paralichthys olivaceous*	18–24	–	HMC**	24.5±0.2	−5.0±0.1	7.7±0.8	6.3±1.6	−21.2±0.1	Folkerts et al. (2024); Oehlert et al. (2024a,b)
*Sparus aurata*	21–26	Nanospheres	ACMC*	50.3	–	–	–	–	Foran et al. (2013)
*Lates calcarifer*, *Lutjanus russellii*, *Sillago maculata*, Sparidae (3 spp.), *Valenciennea immaculata*	24	Ellipsoids, wheatsheafs, rhombohedron, nanospheres	HMC, ACMC, LMC*	15–37	**–**	**–**	**–**	**–**	Salter et al. (2017, 2018)
*Halichoeres bivittatus*, *Thalassoma bifasciatum*, *Lutjanus apodus*, *Ocyurus chrysurus*, *Lutjanus russellii*, *Lutjanus carponotatus*, *Parapercis queenslandica*, *Parapercis australis*, *Sillago sihama*	25	Monocrystalline ellipsoids	HMC*	15–37	**–**	**–**	**–**	**–**	Salter et al. (2017, 2018)
*Albula vulpes*, *Sphyraena barracuda*	25–30	Nanospheres	HMC, ACMC*	25–35	**–**	**–**	**–**	**–**	Perry et al. (2011); Salter et al. (2012)
*Ocyurus chrysurus*, Bothidae, Paralichthyidae, *Lutjanus apodus*, *Epinephelus guttatus*, *Pterois volitans*, *Cephalopholis cruentata*, *Haemulon flavolineatum*, *Mycteroperca bonaci*, *Gerres cinereus*, *Platybelone argalus*	25–30	Monocrystalline ellipsoids	HMC, ACMC*	14–45	**–**	**–**	**–**	**–**	Perry et al. (2011); Salter et al. (2012)
*Sphyraena barracuda*, *Haemulon* sp.	25–30	Small dumbells and wheastsheafs	HMC, ACMC*	30–35	**–**	**–**	**–**	**–**	Perry et al. (2011); Salter et al. (2012)
*Sphyraena barracuda*, *Ocyurus chrysurus*, Bothidae/Paralichthyidae, *Lutjanus apodus*, *Epinephelus guttatus*, *Pterois volitans*, *Cephalopholis cruentata*, *Haemulon flavolineatum*, *Mycteroperca bonaci*, *Gerres cinereus*, *Platybelone argalus*, *Eucinostomus* sp., *Sphoeroides testudineus*, *Pomacanthus arcuatus*, *Acanthostracion quadricornis*, *Sparisoma chrysopterum*	25–30		HMC, HMC, LMC*	2–25	**–**	**–**	**–**	**–**	Perry et al. (2011); Salter et al. (2012)
*Ocyurus chrysurus*	22–29	–	HMC**	22.3±0.8	−3.0±0.9	–	40.4±6.6	23.8±0.9	Folkerts et al. (2024); Oehlert et al. (2024a,b)
*Rachycentron canadum*	22–29	–	–	–	−4.9±0.2	–	–	–	Oehlert et al. (2024a,b)

Data are presented as means±s.e.m. or ranges (*n*). Dashes indicate no prior reports for the compositional characteristics of ichthyocarbonate produced by the listed species at the specified temperature range. Temperature refers to the conditions in which the fish excreted the ichthyocarbonate. Mineralogy was either measured using X-ray diffraction, or inferred based on relationships between crystallite morphology and mol%MgCO_3_ with mineralogy reported previously. ACMC, amorphous calcium magnesium carbonate; HMC, high magnesium calcite; LMC, low magnesium calcite. Measurement and reporting of all other compositional characteristics are defined in the main text.

Some compositional characteristics of blackbelly rosefish ichthyocarbonate were similar between tank-collected and dissection-collected samples, whereas others are distinct (Fig. 4). Excreted ichthyocarbonate was characterized by lower mol%MgCO_3_, C/N ratios and δ^13^C_ichthyo_ values compared with intestinal ichthyocarbonate. Intestinal crystallites collected from both field-collected and laboratory-held fish were not distinguishable, and exhibited a range of morphologies, including <1 µm individual nanospheres and interlocking irregular nanocrystalline material, 0.75 to 1.2 µm ellipsoids and >5 µm spheres (Fig. 5). Ellipsoids (Fig. 5A,E) are frequently composed of high-magnesium calcite (HMC), with magnesium contents typically ranging from ∼20 to 40 mol%MgCO_3_ (Folkerts et al., 2024; Perry et al., 2011; Salter et al., 2017, 2018, 2012; Woosley et al., 2012), consistent with our measurements of mol%MgCO_3_ (Fig. 4). Large spheres (Fig. 5C) are often associated with calcitic mineralogy characterized by 0–15 mol%MgCO_3_, but often less than 5 mol%MgCO_3_ (Salter et al., 2012), a rare but important observation with impacts for ichthyocarbonate fate (Folkerts et al., 2024; Oehlert et al., 2024b). Lack of nanospheres in the tank-collected ichthyocarbonates is consistent with lower mol%MgCO_3_ than the intestinal ichthyocarbonate, and may suggest rapid dissolution of ACMC after ichthyocarbonate excretion, resulting in preferential Mg^2+^ loss (see below for further discussion).

Compositional differences between intestinal and tank-collected ichthyocarbonate may be explained by sequential dissolution of ichthyocarbonate phases with varying stability (Morse et al., 2006), and may also explain some of the heterogeneity observed between intestinal and tank-collected ichthyocarbonate in this study. High solubility of amorphous (Foran et al., 2013) and high-magnesium calcites in ichthyocarbonate (Woosley et al., 2012) may result in rapid (Folkerts et al., 2024; Oehlert et al., 2024b) and preferential dissolution of some phases of ichthyocarbonate after excretion (Morse et al., 2006). Higher mol%MgCO_3_ in intestinal ichthyocarbonate (Fig. 4) is consistent with the interpretation that amorphous phases with higher magnesium contents likely dissolved prior to tank collection. If the relationship between mol%MgCO_3_ and δ^13^C values defined for synthetic HMC (Jimenez-Lopez et al., 2006) can be extrapolated to biogenic HMC, such as ichthyocarbonate, our measurements of δ^13^C_ichthyo_ values further substantiate this interpretation. Not unique to ichthyocarbonate, preferential leaching of less stable Mg-rich phases in planktonic foraminiferal calcites has also been observed to induce changes in mass and density (Johnstone et al., 2011). Such processes can be imagined to shift the tank-collected ichthyocarbonate product towards lower abundances of nanospheres, less amorphous calcium magnesium carbonate (ACMC) and lower mol%MgCO_3_, as discussed above, depending on time of collection. Finally, indistinguishable values of %TOC in intestinal and tank-collected samples indicates that field-collected samples provide relevant compositional information, minimizing the need to collect and rear difficult to maintain mesopelagic fishes in laboratory settings to better develop our understanding of the influence of deepwater fishes on carbon transport.

Compared with ichthyocarbonate produced by other scorpaenids, the crystallite morphology, mineralogy and mol%MgCO_3_ of ichthyocarbonate excreted by blackbelly rosefishes at 6°C is similar despite temperatures ranging from 10 to 25°C (Table 3) (Woosley et al., 2012; Salter et al., 2017, 2018, 2019; Oehlert et al., 2024a,b; Folkerts et al., 2024). *Helicolenus percoides*, for instance, have been observed to produce smaller (<0.7 µm) ellipsoids (Salter et al., 2017, 2018) compared with those produced by the blackbelly rosefish (0.75 to 1.2 µm; Fig. 5). Other confamilials including *Pterois volitans*, *Dendrochirus zebra* and *Scorpaenopsis diabolus* predominantly produce ellipsoids <0.7 µm based on observations of tank-collected material (Salter et al., 2019). However, a more diverse array of crystallite morphologies was produced by the blackbelly rosefish, when intestinal precipitates were compared with tank-collected ichthyocarbonate (Fig. 5). A few explanations have been proposed for the occurrence of variable ichthyocarbonate morphologies. First, a morphogenetic sequence of evolution has been proposed where crystallites evolve predictably with extended growth periods (Perry et al., 2011; Salter et al., 2019, 2012). Second, thermal effects have been proposed to drive variability in crystallite morphology (Ghilardi et al., 2023; Salter et al., 2018) and mol%MgCO_3_ in biogenic carbonates (Chave, 1954), synthetic HMC produced from solutions with varying chemistry (Bertram et al., 1991; Purgstaller et al., 2021) and HMC precipitated from synthetic seawater (e.g. Morse et al., 1997; Mucci, 1987). Comparison of compositional characteristics of ichthyocarbonate produced by blackbelly rosefishes at 6°C with those excreted by confamilials at higher temperatures (10–25°C; Woosley et al., 2012; Salter et al., 2017, 2018, 2019; Oehlert et al., 2024a,b; Folkerts et al., 2024) shows that ichthyocarbonate excreted by fish in warmer waters is characterized by higher mol%MgCO_3_ (Table 3), an observation consistent with recent model results (Ghilardi et al., 2023).

Overall, ichthyocarbonate excreted by blackbelly rosefishes is similar to that produced by shallow dwelling teleosts (Table 3), suggesting that extrapolating measurements of ichthyocarbonate characteristics to global estimates of production, composition and fate or modeling endeavors (Wilson et al., 2009; Ghilardi et al., 2023; Oehlert et al., 2024a; Folkerts et al., 2024) is likely a good first approximation. SEM showed that ichthyocarbonate crystallites excreted by the blackbelly rosefish (Fig. 5) are similar to those of shallow-dwelling fish families tropical and temperate settings (Foran et al., 2013; Ghilardi et al., 2023; Salter et al., 2017, 2019, 2018, 2012, 2014; Walsh et al., 1991; Wilson et al., 2009; Woosley et al., 2012), ruling out a strong effect of depth with pressure <40 atm. Interestingly, blackbelly rosefish are not the only species of marine fish known to produce mixtures of crystallite morphotypes, with members of the Serranidae and Pseudochromidae families producing both ellipsoids and ACMC, and the Blennidae and Pomacentridae families producing ACMC and rhombohedra or spheres, respectively (Salter et al., 2018) (Fig. 5). In terms of their geochemical composition, δ^13^C_ichthyo_ values (−2.9 ‰ on average, *n*=13) approach those of gulf toadfish, a confamilial in the Scorpaenidae family (Table 3) (Oehlert et al., 2024a). Dietary carbon utilization by blackbelly rosefishes was consistent with estimated ranges for four species of shallow dwelling species (Oehlert et al., 2024a), but still below estimated metabolic CO_2_ usage required by physiological mass balance of drinking and total rectal base excretion rates (Genz et al., 2008; Grosell and Genz, 2006; Wilson et al., 1996). Because the metabolic rates of blackbelly rosefishes are ∼24% of what is expected from allometric and thermal relationships and ichthyocarbonate production rates are closer to those based on fish mass and temperature (Fig. 1), the relationship between ichthyocarbonate production and metabolic rate may be higher and thus require higher proportions of seawater DIC. However, assumptions made in model parameterization, as well as the assumption that there is no trans-epithelial fractionation associated with bicarbonate secretion, make these first estimates uncertain. Future studies aimed at resolving these assumptions are warranted.

### Fate of blackbelly rosefish ichthyocarbonate

The particle size of ichthyocarbonate excreted by blackbelly rosefish relative to fish mass is in agreement with the relationship documented from study of shallow water species (Folkerts et al., 2024). Using this recently published relationship, the predicted ichthyocarbonate diameter for blackbelly rosefish is 2.14 mm, similar to the observed average diameter of 2.6 mm. Similarly, the specific gravity of ichthyocarbonate excreted by blackbelly rosefish of 1.234 g cm^−3^ is within the range (1.23–1.34 g cm^−3^) reported for ichthyocarbonate excreted by shallow water species (Folkerts et al., 2024). The observed dissolution rates of 205 µeqv HCO_3_^−^ g^−1^ h^−1^ for ichthyocarbonate excreted by blackbelly rosefish is higher than dissolution rates of 28 to 154 µeqv HCO_3_^−^ g^−1^ h^−1^ reported for shallow water species at the same aragonite saturation state (Folkerts et al., 2024). This difference is expected considering that the dissolution rates from the shallow water species were measured at 20°C in contrast to the 6°C used for the blackbelly rosefish, and that dissolution rates of CaCO_3_ generally are higher at colder temperatures (Adkins et al., 2021; Naviaux et al., 2019). Mol%MgCO_3_ did not correlate with dissolution rates of ichthyocarbonate produced by shallow water species (Folkerts et al., 2024), and the 28 mol%MgCO_3_ found in ichthyocarbonate excreted by blackbelly rosefish is within the range reported for shallow water species (23–32%) and therefore does not predict the high dissolution rate. Removal of external organic matter coatings on ichthyocarbonate has been shown to greatly enhance dissolution rates (Oehlert et al., 2024b) and blackbelly rosefish ichthyocarbonate has lower TOC (3.8%) than any other species examined to date (5.5–40%) (Oehlert et al., 2024a). Thus, it is tempting to suggest that lower TOC in blackbelly rosefish, or perhaps a difference in the composition of the organic coatings, may explain the higher dissolution rates. Comparisons of the proteinaceous matrix associated with ichthyocarbonate revealed some differences in the type of proteins found across species (Schauer et al., 2018a). Given that the proteinaceous matrix influence ichthyocarbonate formation (Schauer et al., 2016) and that different proteins contribute differently across species (Schauer and Grosell, 2017; Schauer et al., 2018b), it seems likely that differences in the proteinaceous matrix may either directly or indirectly influence dissolution rates.

The ultimate fate of ichthyocarbonate is a product of sinking rate, which can be estimated from size and specific gravity using Stokes’ law and measured dissolution rates. Applying a recent model designed to examine the depth of full dissolution for ichthyocarbonate produced by three shallow water species (Folkerts et al., 2024), we evaluated blackbelly rosefish ichthyocarbonate. The blackbelly rosefish is a benthic fish, and ichthyocarbonate from this species therefore reaches the sediment immediately after excretion. However, the model also predicts time to complete dissolution, which in the case of blackbelly rosefish ichthyocarbonate excreted at ∼400 m water depth in the Straits of Florida, is 6 days. Using this model, we can also deduce the amount dissolved in the holding tanks during the 24-h period allowed for ichthyocarbonate to accumulate. Assuming that the average ‘age’ of ichthyocarbonate at the time of collection every 24 h is 0.5 days, ∼8% of ichthyocarbonate dissolves before collection. This modest loss of ichthyocarbonate may account for the observed absence of nanospheres, inferred to be ACMC based on published correlations (Salter et al., 2018, 2019), and significant changes in mol%MgCO_3_ observed between intestinal and tank-collected ichthyocarbonate discussed above.

Applying the observations from blackbelly rosefish ichthyocarbonate size, specific gravity and dissolution rate to ichthyocarbonate excreted by a hypothetical mesopelagic at a depth of 400 m, the sinking ichthyocarbonate would persist to a depth of 1331 m prior to complete dissolution. Further, many mesopelagic fishes vertically migrate from 200 m at night to 1000 m during the day (Govindarajan et al., 2023; Salvanes and Kristoffersen, 2001), presumably excreting at least part of their ichthyocarbonates at 1000 m, translating to a depth of full dissolution >1900 m. The dissolution depth estimates reported are likely overestimates, because temperature and Ω were held constant in these estimates (6°C and 1, respectively), but the influence of these factors is unknown, but should decrease with depth and affect both dissolution and sinking rates (Folkerts et al., 2024). Blackbelly rosefish are also larger than most mesopelagic fish and therefore produce large ichthyocarbonates, which strongly influences fate, and the majority of globally produced ichthyocarbonates will have a diameter of ∼0.25 mm (Folkerts et al., 2024). If released at the sea surface, these smaller ichthyocarbonates are anticipated to dissolve fully in the top 100 m of global oceans (Folkerts et al., 2024). However, if we assume that small mesopelagic fish (Salvanes and Kristoffersen, 2001) excrete ichthyocarbonates of 0.25 mm in diameter at 1000 m, and assume specific gravity and dissolution rates as defined for blackbelly rosefish ichthyocarbonate, they would persist for ∼0.5 days and travel not much deeper than the depth of release, adding to the deepwater alkalinity pool. However, vertically migrating mesopelagic fishes feed at shallower depths (∼200 m) and so the carbon in the mineral phase of ichthyocarbonate contains important quantities of dietary carbon, which is endogenous carbon of metabolic origin derived from ingested organic matter. In addition, at least part of the ichthyocarbonate-associated organic material would presumably be released at depths up to or beyond 1000 m. The consequence of the vertical migrations by mesopelagic fishes is thus potentially a rapid transfer of carbon in the form of both inorganic and organic carbon from shallower to deeper depths. Notably, this vertical migration, and with it, a vertical carbon flux, often traverses the thermocline to depths where carbon sequestration occurs over the timescale of centuries to millennia (Lampitt et al., 2008; Saba et al., 2021). Considering that the majority of global fish biomass is ascribed to mesopelagic fish (Irigoien et al., 2014; Oehlert et al., 2024a; Proud et al., 2019), it is critical to determine whether mesopelagic fish preferentially excrete ichthyocarbonate at the shallowest or deepest depths of their migration. Ichthyocarbonate release by individual fish is episodic and under sophisticated endocrine control (Ruhr et al., 2014, 2015, 2018, 2016), but we know very little about the timing and frequency of release, offering an important area for future research.

### Summary

Deep dwelling blackbelly rosefish residing at 6°C precipitate ichthyocarbonate in their intestine with production rates, morphology and composition similar to those produced by shallow water species. Further, intestinal fluid chemistry resembles that of shallow water species. Although angling stress may affect both intestinal fluid and blood chemistry, ichthyocarbonates obtained from the intestine show no differences, with the exception of δ^13^C_org_ values, between field-collected and lab-acclimated fish, ruling out a pressure effect on ichthyocarbonate formation, at least for pressures up to 40 atm. Thus, based on just the single species studied here, it appears that what we know about ichthyocarbonate formation and excretion from shallow water species is representative for fish residing in deeper and colder environments, offering support for the use of more convenient model species for understanding of the role of mesopelagic fish in global ichthyocarbonate production and fate. In contrast, however, excreted ichthyocarbonates show different mol%MgCO_3_ and morphology than corresponding samples from the intestine, likely owing to rapid differential dissolution of ichthyocarbonate fractions post excretion. The latter means that caution should be applied when basing conclusions on analyses performed on ichthyocarbonates collected from the intestine.
